# Serum Lipase Amylase Ratio as an Indicator to Differentiate Alcoholic From Non-alcoholic Acute Pancreatitis: A Systematic Review and Meta-Analysis

**DOI:** 10.7759/cureus.35618

**Published:** 2023-02-28

**Authors:** Nishith M Ekka, Archana D Kujur, Rishi Guria, Mrityunjay Mundu, Brajesh Mishra, Sulakshana Sekhar, Amit Kumar, Jay Prakash, Hirendra Birua

**Affiliations:** 1 Surgery, Rajendra Institute of Medical Sciences, Ranchi, IND; 2 Pharmacology and Therapeutics, Rajendra Institute of Medical Sciences, Ranchi, IND; 3 Internal Medicine, Rajendra Institute of Medical Sciences, Ranchi, IND; 4 Pulmonary Medicine, Rajendra Institute of Medical Sciences, Ranchi, IND; 5 General Surgery, Rajendra Institute of Medical Sciences, Ranchi, IND; 6 Laboratory Medicine, Rajendra Institute of Medical Sciences, Ranchi, IND; 7 Critical Care Medicine, Rajendra Institute of Medical Sciences, Ranchi, IND; 8 Pediatric Surgery, Rajendra Institute of Medical Sciences, Ranchi, IND

**Keywords:** alcoholic pancreatitis, lipase/amylase ratio, diagnosis, l/a ratio, alcoholic, pancreatitis

## Abstract

A lipase/amylase (L/A) ratio of more than three may be a tool for differentiating alcoholic pancreatitis from non-alcoholic pancreatitis. We conducted a systematic literature review to identify published studies. A thorough data search of various databases was conducted using keywords. Study quality was assessed using the Quality Assessment of Diagnostic Accuracy Studies-2 survey. Data were extracted under the following headings: country, sample size, baseline characteristics, specificity, and sensitivity of the L/A ratio. Studies were analyzed using a bivariate random-effects model, and the sensitivity and specificity of the L/A ratio were pooled separately. Summary receiver operating characteristic (SROC) curves were plotted using the hierarchical method. A total of nine studies with 1,825 patients were identified for inclusion. SROC showed estimates of the area under the curve to be 0.75 (confidence interval (CI) = 0.71-0.79). Forest plots for sensitivity and specificity showed pooled estimates of sensitivity to be 74% (95% CI = 62-83%) while that of specificity was 63% (95% CI = 47-77%). The pooled diagnostic odds ratio was estimated to be 5 (95% CI = 3-9), the pooled positive likelihood ratio was estimated at 2.0, and the pooled negative likelihood ratio was estimated to be 0.41. We concluded that an L/A ratio of more than 3 has moderate accuracy for the diagnosis of alcoholic pancreatitis.

## Introduction and background

Acute pancreatitis is one of the most common gastrointestinal causes of hospitalization in the United States [1]. Various studies have reported high mortality rates of up to 21% [2,3]. With 618,862 new cases, India had the most number of incident cases in the world in 2019 [4]. Acute pancreatitis is defined using the revised Atlanta criteria by meeting two of the three following criteria: abdominal pain consistent with acute pancreatitis, serum lipase or amylase activity at least three times greater than the upper limit of normal, and characteristic findings of acute pancreatitis on contrast-enhanced CT, MRI, or transabdominal ultrasonography [5]. Alcohol is the second most common cause of acute pancreatitis after gallstones [6]. Although there are no universally accepted criteria to assign alcohol as an etiological factor, many experts have defined it as consumption of over 50-80 g (four to seven drinks per day) [7]. In 1991, Gumaste et al. proposed a new index, the lipase/amylase (L/A) ratio, to distinguish acute episodes of alcoholic from non-alcoholic acute pancreatitis [8]. Since then, several studies have been conducted to validate these findings [9-11]. In the literature search, we did not find a meta-analysis assessing the diagnostic accuracy of the L/A ratio to differentiate alcoholic from non-alcoholic causes of acute pancreatitis. Answering the question of whether an L/A ratio of three or more can correctly diagnose alcoholic pancreatitis will help clinicians ascertain the cause and accordingly advise lifestyle modifications. Thus, we performed this systematic review and meta-analysis, the proposal for which was prospectively registered in Prospero (ID: CRD42022306082) [12].

## Review

Search strategy

A thorough data search of PubMed including PubMed Central, Google Scholar, and Cochrane databases was undertaken to identify studies with a focus on the research question in the Patient, Intervention, Comparison, and Outcome (PICO) format, that is, in patients of acute pancreatitis as defined by the Atlanta criteria, does a lipase/amylase ratio of more than three, identifies alcoholic pancreatitis more accurately than a ratio of less than three? Alcohol was assigned as a causative factor if the patient had a history of alcohol consumption over 50-80 g/day. Language restrictions were not imposed. The bibliography of all articles was also checked for eligible articles unidentified in the database search. MeSH search was performed using keywords (((alcoholic) AND (pancreatitis)) AND (lipase/amylase ratio)) AND (lipase amylase ratio)) AND (diagnosis). Studies were scrutinized for eligibility using the following inclusion criteria: (1) participants were diagnosed with acute pancreatitis; (2) all studies that reported the L/A ratio in alcoholic and non-alcoholic groups; (3) sensitivity and specificity and cut-off values mentioned; and (4) both prospective and retrospective studies were included. Two investigators independently selected the eligible articles. All disagreements between the investigators were resolved by dialogue.

Data extraction and quality assessment

**Figure 1:**
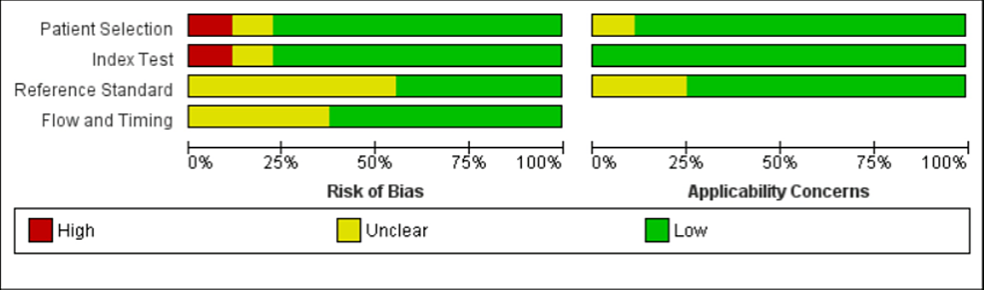
Summary of quality assessment. Risk of bias and concerns regarding applicability for studies included in the meta-analysis, according to the Quality Assessment of Diagnostic Accuracy Studies-2 tool.

Two different investigators, NE and AK, simultaneously extracted data under the following headings: country of study, the sample size for each group, baseline characteristics of subjects, specificity and sensitivity of the L/A ratio for diagnosis of alcoholic acute pancreatitis, and the number of true-positive and false-positive rates. The Quality Assessment of Diagnostic Accuracy Studies-2 tool was used for quality assessment. The risk of bias summary and the graph were generated using Cochrane RevMan 5.4 software (Figure 1).

Statistical analysis

Studies were analyzed using a bivariate random-effects model, and the sensitivity and specificity of the L/A ratio were pooled separately. Summary receiver operating characteristic (SROC) curves were plotted using the hierarchical method. The computation was done by using Stata version 17 software (StataCorp., College Station, TX, USA). The heterogeneity among studies was tested using the chi-square-based Q test. Furthermore, the percentage of interstudy variation in the total variation was evaluated using the I^2^ value. A p-value <0.05 and/or I^2^ >50% suggested significant heterogeneity among studies. Publication bias was evaluated using the Deeks’ funnel plot.

Results

Study Selection

**Figure 2:**
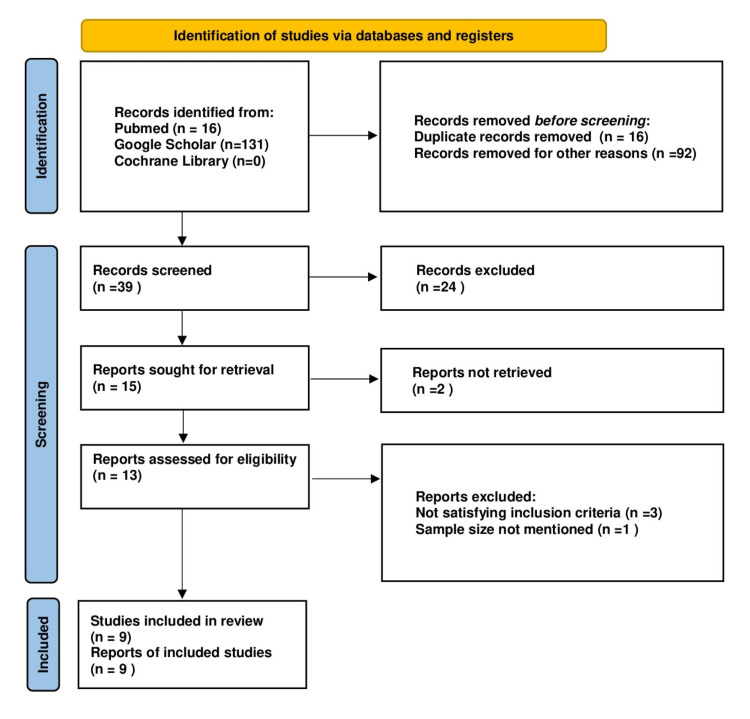
PRISMA 2020 flow diagram illustrating the electronic database searches and selection of studies in the meta-analysis based on the PRISMA 2020 statement. Ninety-two studies did not match our research question on abstract review, while 24 studies were removed after full-text review and did not satisfy the quality assessment. PRISMA = Preferred Reporting Items for Systematic Reviews and Meta-Analyses

A total of 147 articles were identified through a database search, and after duplicate and records removal for other reasons, 39 articles were selected for abstract screening. A total of 13 articles were considered eligible for full-text review. After excluding three articles for not satisfying the inclusion criteria and one article for having a lack of data for sample size, nine articles were included with a total of 1,825 subjects (Table 1). The procedure for selection is shown in a flowchart based on the Preferred Reporting Items for Systematic Reviews and Meta-Analyses 2020 statement [13] (Figure 2).

**Table 1:** Various characteristics of studies. Author, year, country, study type, the total sample, mean age, cut-off, sensitivity, and specificity of various included studies.

Study	Year	Country	Type of study	Mean age	Sample size	Cut-off	Sensitivity	Specificity
Gumaste et al. [8]	1991	USA	Prospective	45.1	30	3	0.83	0.88
Sadowski and Sutherland [9]	1993	Canada	Retrospective	48.5	162	3	0.70	0.60
Pezzilli et al. [10]	1993	Italy	Prospective	64	66	3	0.29	0.67
Mimoz et al. [11]	1993	France	Prospective	57	51	2	0.91	0.40
Devanath et al. [14]	2009	India	Retrospective	52.2	1132	3	0.76	0.64
Tenner et al. [15]	1992	USA	Retrospective	45	47	3	0.59	0.78
Pacheco et al. [16]	2007	Portugal	Prospective	42.7	38	3	0.67	0.76
Singh et al. [17]	2020	India	Prospective	41.5	52	3	0.71	0.90
Chang et al. [18]	2005	Taiwan	Prospective	53.6	247	2	0.93	0.18

Three studies were excluded for not satisfying the inclusion and exclusion criteria. Ansari et al. did not mention the sensitivity or specificity of the L/A ratio [19]. Haque et al. mentioned the L/A ratio as the mean and standard deviation in alcoholic and non-alcoholic groups but did not calculate its sensitivity or specificity [20]. Kumar et al. also mentioned the mean of the L/A ratio but did not calculate the sensitivity or specificity [21].

**Figure 3:**
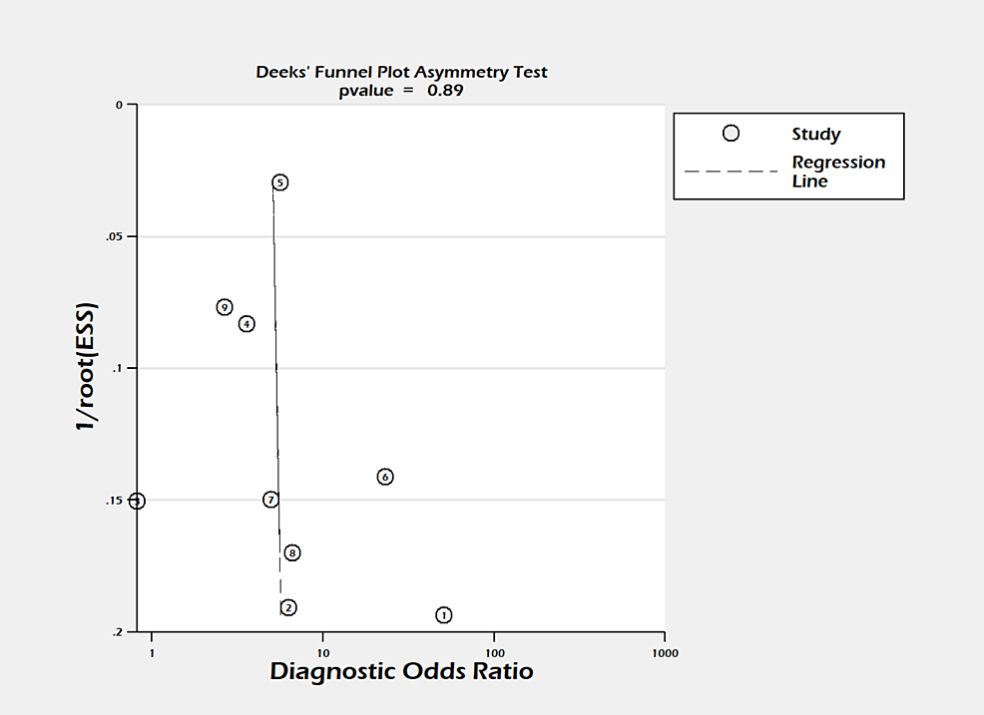
Deeks’ funnel plot. Funnel plot for assessment of publication bias in studies evaluating the lipase/amylase ratio in differentiating alcoholic pancreatitis. Deeks’ funnel plot asymmetry test (P = 0.89). ESS = effective sample size

Diagnostic Accuracy of the Lipase/Amylase Ratio

**Figure 4:**
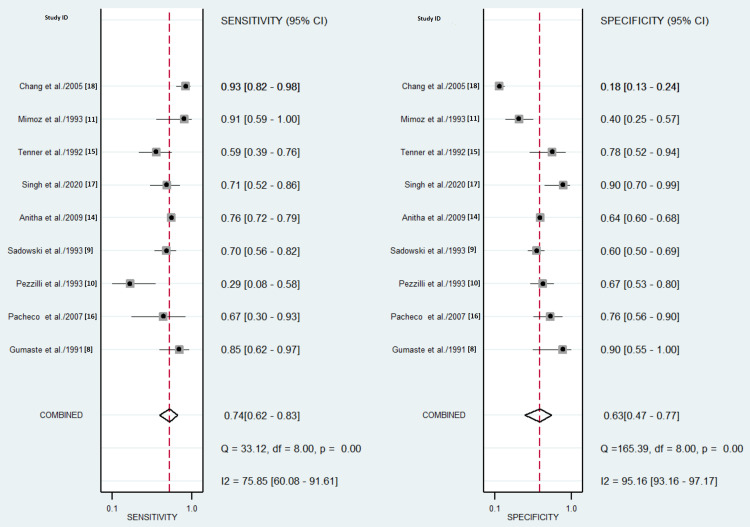
Forest plots of sensitivity and specificity. Sensitivity = 74% (95% CI = 62-83%, I^2^ = 75.85%, p = 0.001), specificity = 63% (95% CI = 47-77%, I^2^ = 95.16%, p < 0.001).

Forest plots showed sensitivity ranging from 28.6% to 92.6% and specificity ranging from 18 to 90%, as shown in Figure 4. Pooled estimates of sensitivity were observed to be 74% (95% confidence interval (CI) = 62-83%, I^2^ = 75.85%, p = 0.001) while that of specificity was 63% (95% CI = 47-77%, I^2^ = 95.16%, p < 0.001) (Figure 4).

The diagnostic odds ratio ranged between 0.82 and 51, and the pooled diagnostic odds ratio was estimated to be 5 (95% CI = 3-9). A positive likelihood ratio among studies was observed to be between 1.12 and 8.50, with an observed pooled positive likelihood ratio of 2.0 (95% CI = 1.4-2.9). The negative likelihood ratio ranged from 0.17 to 1.06, and pooled negative likelihood ratio was estimated to be 0.41 (95% CI = 0.30-0.56).

The Fagan Nomogram

**Figure 5:**
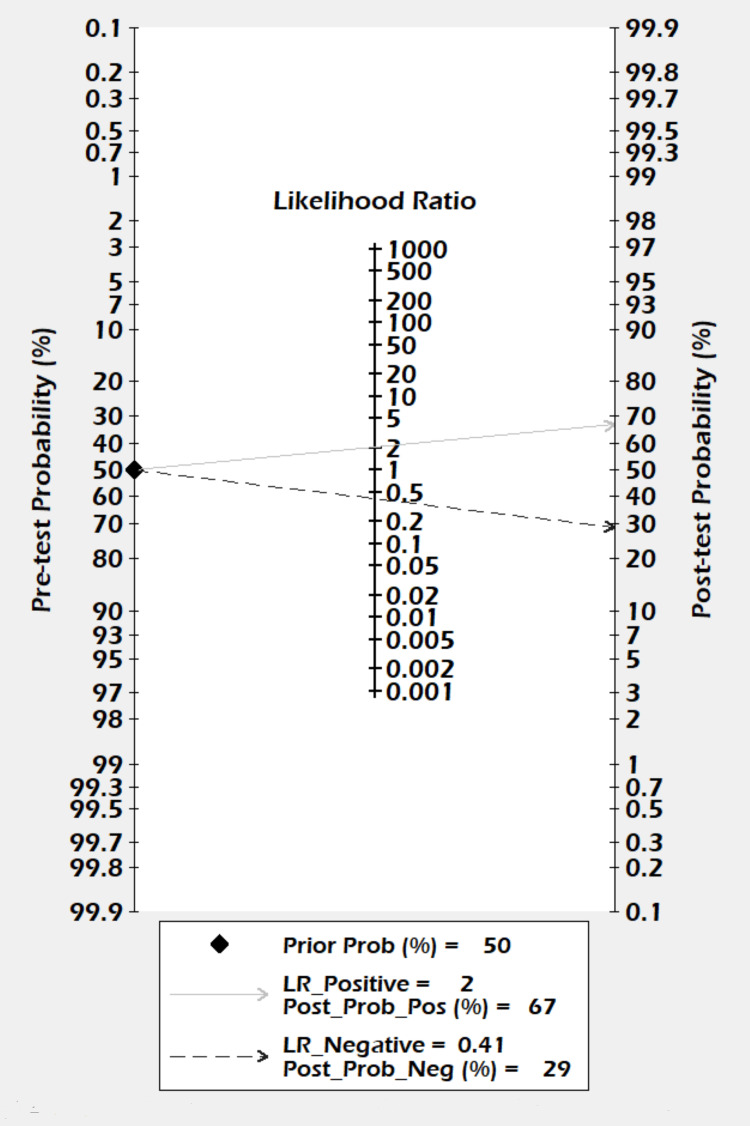
Fagan nomogram for the lipase/amylase ratio in differentiating alcoholic pancreatitis. The prior probability was 50%. The post-test probability was 67% following a positive result and 29% following a negative result. LR = likelihood ratio; Prob = probablity; Pos = positive; Neg = negative

The Fagan nomogram showed that a positive result increased the post-test probability of alcoholic pancreatitis from 50% to 67%, whereas a negative test decreased the post-test probability of alcoholic pancreatitis to 29% (Figure 5).

Summary Receiver Operating Characteristic Curve Analysis

**Figure 6:**
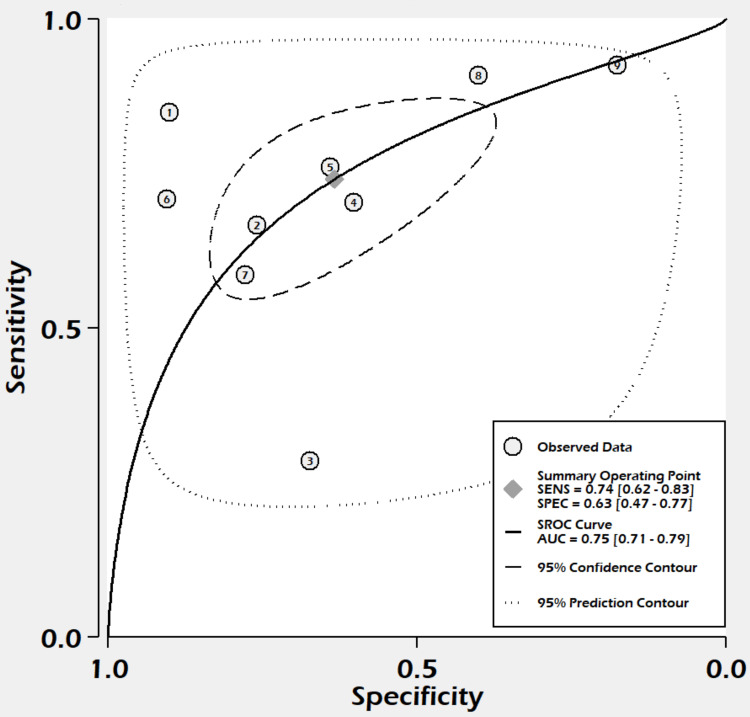
SROC curve. AUC was estimated to be 0.75 (95% CI = 0.71-0.79, SE = 0.04). SE = sample entropy; SENS = sensitivity; SPEC = specificity; SROC = summary receiver operating curve; AUC = area under the curve

With sensitivity, the true-positive rate on the y-axis, and specificity, the false-positive rate on the x-axis, a graph was plotted showing the relationship between the true-positive rate and the false-positive rate of the L/A ratio at different thresholds. SROC is shown in Figure 6, and the area under the curve was estimated to be 0.75 (95% = CI 0.71-0.79, sample entropy (SE) = 0.04) (Figure 6).

Meta-Regression

**Figure 7:**
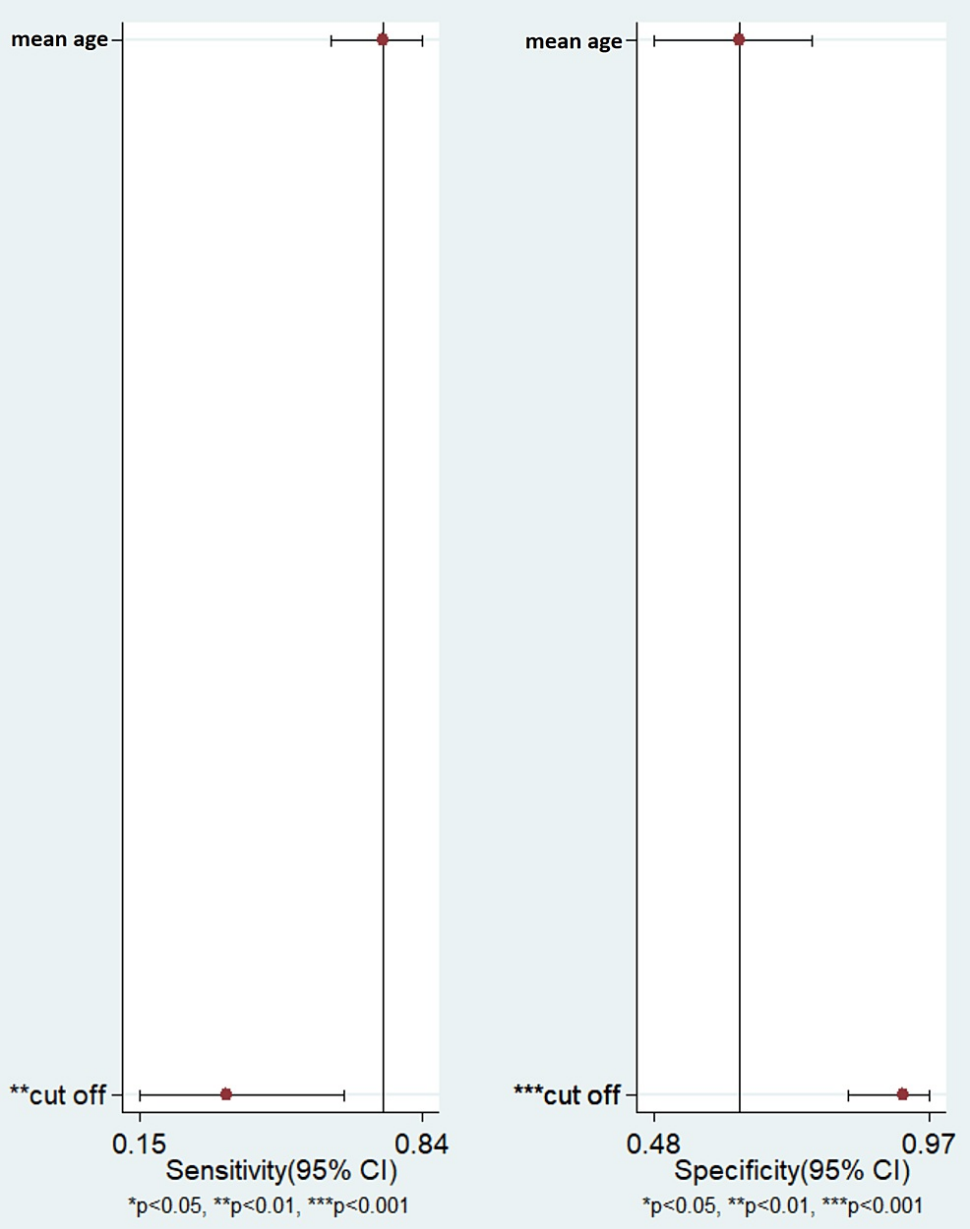
Meta-Regression Analysis. Results adjusted for mean age and cut-off value. CI= Confidence Interval

In the meta-regression analysis, we observed that the mean age in the study did not explain the heterogeneity, while the cut-off value may have significantly affected the heterogeneity observed in the pooled sensitivity and pooled specificity for the L/A ratio for differentiating alcoholic from non-alcoholic pancreatitis (Figure 7).

Discussion

Summary of Findings

In this review, we observed that an L/A ratio of three or more, although not excellent, is an acceptable indicator of alcoholic pancreatitis. Patients with alcoholic pancreatitis have twice the chance of having an L/A ratio of three or more. Pooled sensitivity and specificity were estimated to be 75% and 56%, respectively.

Applicability of Evidence

Since its first mention in 1878, the association between alcohol abuse and pancreatic injury is well established [22]. The etiology of pancreatitis is attributed to alcohol if the patient has a history of alcohol abuse. There is no biochemical marker that can distinguish alcoholic from non-alcoholic pancreatitis. To date, there is no way to attribute the cause of pancreatitis to alcohol. History from the patient or relatives is often misguiding [23]. Gumatse first reported that an L/A ratio of three or more can differentiate between alcoholic and non-alcoholic pancreatitis [8]. Other studies that followed have reported contradicting results [9-11,14]. This review observed that the L/A ratio can diagnose and differentiate alcoholic pancreatitis with a fair degree of precision, and in the absence of better biochemical indicators it can be used in clinical practice.

Quality of Evidence

We reviewed a total of nine studies with 1,825 participants. Most studies included consecutive patients and defined a pre-set threshold value. Three studies [9,14,15] out of the nine were retrospective while the rest were prospective [8,10,11,16-18]. Six studies reported sensitivity above 70% while four studies reported specificity over 70%. There is a high degree of inconsistency in the findings of these studies. We observed the existence of heterogeneity in our study, with I^2^ of more than 50%. This is likely due to the threshold effect.

Study limitations

The literature review was thorough but studies from only a few databases could be reviewed. The presence of significant heterogeneity in the study could be a source of bias. Moreover, we could not compare more covariates, which could be present.

## Conclusions

In this study, we found that an L/A ratio of more than three had moderate accuracy for the diagnosis of alcoholic pancreatitis. In the absence of better diagnostic modalities, the L/A ratio can be used in clinical practice to attribute the cause of pancreatitis to alcoholism and persuade patients to abstain. For better evidence, well-designed prospective studies are needed.
